# Open lung approach with low tidal volume mechanical ventilation attenuates lung injury in rats with massive brain damage

**DOI:** 10.1186/cc13813

**Published:** 2014-04-02

**Authors:** Joerg Krebs, Charalambos Tsagogiorgas, Paolo Pelosi, Patricia RM Rocco, Maximilia Hottenrott, Carsten Sticht, Benito Yard, Thomas Luecke

**Affiliations:** 1 Department of Anaesthesiology and Critical Care Medicine, University Medical Centre Mannheim, Medical Faculty Mannheim of the University of Heidelberg, Mannheim, Germany, Theodor-Kutzer Ufer, 68165 Mannheim, Germany; 2 Department of Surgical Sciences and Integrated Diagnostics, IRCCS AOU San Martino -IST, University of Genoa, Genoa, Italy; 3 Laboratory of Pulmonary Investigation, Carlos Chagas Filho Biophysics Institute, Federal University of Rio de Janeiro, Av. Carlos Chagas Filho, s/n, Bloco G-014, Ilha do Fundão, 21941-902 Rio de Janeiro, RJ, Brazil; 4 Medical Research Center, Medical Faculty Mannheim of the University of Heidelberg, Mannheim, Germany, Theodor-Kutzer Ufer, 68165 Mannheim, Germany; 5 Department of Medicine, University Medical Center Mannheim, Medical Faculty Mannheim of the University of Heidelberg, Mannheim, Germany, Theodor-Kutzer Ufer, 68165 Mannheim, Germany

## Abstract

**Introduction:**

The ideal ventilation strategy for patients with massive brain damage requires better elucidation. We hypothesized that in the presence of massive brain injury, a ventilation strategy using low (6 milliliters per kilogram ideal body weight) tidal volume (V_T_) ventilation with open lung positive end-expiratory pressure (LV_T_/OLPEEP) set according to the minimal static elastance of the respiratory system, attenuates the impact of massive brain damage on gas-exchange, respiratory mechanics, lung histology and whole genome alterations compared with high (12 milliliters per kilogram ideal body weight) V_T_ and low positive end-expiratory pressure ventilation (HV_T_/LPEEP).

**Methods:**

In total, 28 adult male Wistar rats were randomly assigned to one of four groups: 1) no brain damage (NBD) with LV_T_/OLPEEP; 2) NBD with HV_T_/LPEEP; 3) brain damage (BD) with LV_T_/OLPEEP; and 4) BD with HV_T_/LPEEP. All animals were mechanically ventilated for six hours. Brain damage was induced by an inflated balloon catheter into the epidural space. Hemodynamics was recorded and blood gas analysis was performed hourly. At the end of the experiment, respiratory system mechanics and lung histology were analyzed. Genome wide gene expression profiling and subsequent confirmatory quantitative polymerase chain reaction (qPCR) for selected genes were performed.

**Results:**

In NBD, both LV_T_/OLPEEP and HV_T_/LPEEP did not affect arterial blood gases, as well as whole genome expression changes and real-time qPCR. In BD, LV_T_/OLPEEP, compared to HV_T_/LPEEP, improved oxygenation, reduced lung damage according to histology, genome analysis and real-time qPCR with decreased interleukin 6 (IL-6), cytokine-induced neutrophil chemoattractant 1 (CINC)-1 and angiopoietin-4 expressions. LV_T_/OLPEEP compared to HV_T_/LPEEP improved overall survival.

**Conclusions:**

In BD, LV_T_/OLPEEP minimizes lung morpho-functional changes and inflammation compared to HV_T_/LPEEP.

## Introduction

Mechanical ventilation is a lifesaving treatment in patients suffering from massive brain damage caused by hemorrhage, ischemic stroke or severe traumatic brain injury [1]. Pulmonary dysfunction is the most frequent extracerebral complication in neurological patients undergoing mechanical ventilation [2] and acute respiratory distress syndrome (ARDS) is recognized as an independent predictor of poor outcome [3]. Pulmonary dysfunction associated with acute brain damage has long been attributed only to a greater increase in sympathetic activity with pulmonary venoconstriction and higher capillary permeability, but recent studies also reported the role of a systemic inflammatory response with pulmonary infiltration of neutrophils, cytokine release and endothelial dysfunction triggered by an initial sympathetic discharge [3,4]. Other factors may also be involved in the complex cross-talk between brain and lungs, like excessive inflammation due to the brain damage-induced impairment of the parasympathetic nervous system [5] and hence, the loss of the protective cholinergic anti-inflammatory pathway [6].

It has been clearly shown in experimental [7,8] as well as clinical settings that mechanical ventilation itself might induce [9,10] or worsen [11] existing lung damage (ventilator-associated lung injury, VALI). VALI is caused by alveolar over-distension and repetitive opening and closing of atelectatic lung regions, respectively [12]. Both conditions ultimately lead to parenchymatous inflammation and, consecutively, ARDS [7]**,** which may cause dysfunction in downstream organs, such as the small bowel, kidney [13] or the brain itself [14].

Apart from limiting the applied tidal volume (V_T_) to 6 mL/kg ideal body weight and keeping end-inspiratory plateau pressure (Pinsp) below 30 cmH_2_O [11], the ideal ventilatory pattern in ARDS is not yet characterized. As an additional component of ventilation strategy for patients with ARDS, the use of recruitment maneuvers in addition to adequate PEEP set during a decremental PEEP trial targeting maximum compliance [15] has been suggested, albeit scientific proof is lacking [16].

In the setting of massive brain damage, high PEEP strategies are thought to compromise cerebral perfusion pressure (CPP) and are therefore not routinely applied in neurocritical care [2]. However, neither the ideal ventilation strategy for patients with massive brain damage nor the underlying biological mechanisms leading to acute respiratory dysfunction are clearly defined.

We hypothesized that in the presence of massive brain injury, a ventilation strategy using low V_T_ and PEEP set according to the minimum elastance of the respiratory system (LV_T_/OLPEEP) may improve lung morphofunction and minimize VALI more effectively than a ventilation regimen using high V_T_ and low PEEP (HV_T_/LPEEP).

## Materials and methods

The study was approved by the Institutional Review Board for the care of animal subjects (University of Heidelberg, Mannheim, Germany). All animals received humane care in compliance with the “Principles of Laboratory Animal Care” formulated by the National Society for Medical Research and the “Guide for the Care and Use of Laboratory Animals” prepared by the U.S. National Academy of Sciences.

### Animal preparation and experimental protocol

A total of 28 specific pathogen-free male Wistar rats (450 to 500 g) housed in standard conditions with food and water *ad libitum* were anesthetized by intraperitoneal (i.p.) injection of ketamine hydrochloride (50 mg/kg; Ketanest 10%®, Pfizer, Karlsruhe, Germany) and xylazine (2 mg/kg; Rompun®, BayerVital, Leverkusen, Germany). Anesthesia was maintained with intravenous ketamine throughout the experiment. The femoral artery and the femoral vein were cannulated with polyethylene catheter tubing (PE-50, neoLab Heidelberg, Germany).

The arterial line was used for continuous monitoring of heart rate (HR) and mean arterial pressure (MAP) and to collect intermittent blood samples (100 μl) for blood-gas analysis (Cobas b121, Roche Diagnostics GmbH, Wien, Austria).

The animals were then put in supine position, tracheotomized, intubated with a 14G polyethylene tube (Kliniject, KLINIKA Medical GmbH, Usingen, Germany) and mechanically ventilated with a neonatal respirator (Babylog 8000, Draeger, Luebeck, Germany) in pressure-controlled mode with a PEEP of 2 cm H_2_O, an inspiratory:expiratory ratio (I:E) of 1:1 and fraction of inspired oxygen (FiO_2_) of 0.5. FiO_2_ was maintained constant throughout the entire experimental period. Pinsp was adjusted to maintain a V_T_ of 6 mL/kg body weight. A variable respiratory rate (RR) of 90 to 110 breaths/minute was applied to maintain a PaCO_2_ value within physiological range. A catheter with a protected tip was inserted in the esophagus for measurement of esophageal pressure (Pes). Proper catheter position was confirmed in all animals, as described previously [17]. Additional intravenous fluid boluses of balanced electrolyte solution (Deltajonin, Deltaselect GmbH, Muenchen, Germany) were given, aiming to maintain a MAP of at least 60 mmHg. The amount of fluid administered and the weight gain after the six-hour experimental period were recorded in each animal. No catecholamines were administered. Body temperature was maintained between 37°C and 38.5°C with a heating pad.

### Experimental protocol

Upon completion of the instrumentation, animals were allowed to stabilize for 15 minutes and were then randomly allocated to one of two groups to receive low V_T_ (6 mL/kg body weight) and open lung PEEP ventilation (LV_T_/OLPEEP, n = 14) or high V_T_ (12 mL/kg body weight) and low PEEP ventilation (HV_T_/LPEEP, n = 14) (Baseline). In the LV_T_/OLPEEP group, a recruitment maneuver, applied as continuous positive airway pressure of 25 cm H_2_O for 40 seconds followed by a decremental PEEP trial started at 10 cm H_2_O, was performed. Pinsp was adjusted to deliver a V_T_ of 6 mL/kg body weight followed by a full measurement of respiratory system mechanics, gas exchange and hemodynamics. PEEP was then reduced in steps of 2 cm H_2_O and changes in static elastance of the respiratory system (Estat,RS) were measured after a 10-minute equilibration period. PEEP was reduced until the Estat,RS no longer decreased. PEEP at minimum Estat,RS was defined as “open lung PEEP” (OLPEEP) as previously described [4,5]. Animals were then re-recruited and LV_T_/OLPEEP was applied throughout the experimental period. All other ventilator settings remained unchanged. Animals in each treatment group were then further randomized into two subgroups according to the induction of massive brain damage (BD) or not (NBD). Brain damage was induced with a 4.7 F Fogarty catheter (Cardinal Health, Dublin, OH, USA) inserted into the epidural space through an occipital burr hole and then gradually inflated over a one-minute period with 750 μl of saline [18,19]. Subdural or intracerebral placement of the catheter was avoided to prevent extracranial herniation of brain tissue. Massive brain damage was verified by the presence of the Cushing response during balloon inflation and dilated, fixed pupils without photomotor reflex [19]. Animals in the NBD groups received no trepanation. All four subgroups (NBD and BD with LV_T_/OLPEEP or HV_T_/LPEEP) were ventilated for six hours. Hemodynamics (MAP and HR) were recorded online and blood gas analysis was performed hourly. At the end of the experiment, the respiratory system, lung and chest wall static elastance, gas exchange and hemodynamics were assessed as well as body weight. Immediately after the measurement of respiratory mechanics (END), 1,000 IU of heparin were injected intravenously and a laparotomy was done. The trachea was clamped at 5 cmH_2_O PEEP in all groups to standardize pressure conditions. The abdominal aorta and vena cava were sectioned, yielding a massive hemorrhage that quickly killed the animals. Lungs were removed *en bloc*. The right lungs were quick frozen in nitrogen for mRNA extraction and GeneArray analysis. The left lungs were immersed in 4% formalin and embedded in paraffin.

#### Respiratory system, lung and chest wall mechanics

Tracheal (Ptrach) and esophageal pressures (Pes) were recorded during three to four seconds of airway occlusion at end-expiration and end-inspiration. Estat,RS was computed as Estat,RS = ΔPtrach/V_T_, where ΔPtrach is the difference between end-inspiratory and end-expiratory tracheal pressure. Static elastance of the chest wall (Estat,CW) was computed as ΔPes/V_T_, where ΔPes is the difference between end-inspiratory and end-expiratory esophageal pressure. Static lung elastance (Estat, L) was calculated as (Estat,L = Estat,RS - Estat,CW) [7,8].

#### Histological examination

Paraffin-embedded specimens were cut into 4 μm-thick slices and stained with hematoxylin and eosin. Morphological examination was performed in a blinded fashion by two investigators, using a conventional light microscope at ×100 magnification across 10 random, non-coincident fields of view. A five-point semiquantitative severity-based scoring system was used as previously described [7]. Pathological findings were graded as absent = 0, slight = 1, moderate = 2, high = 3 and severe = 4. The amount of intra- and extra-alveolar hemorrhage, intra-alveolar edema, inflammatory infiltration of the interalveolar septa and airspace, atelectasis and over-inflation were rated. The scoring variables were added and a histological total lung injury score per slide was calculated.

#### Affymetrix whole transcript expression analysis and confirmatory qPCR

Total RNA of right lung tissue was extracted and DNase treatment was carried out. RNA concentration was assessed by 260/280 measurements using the Infinite® 200 PRO NanoQuant (Tecan Group Ltd., Maennedorf, Switzerland), and RNA integrity was measured by Agilent Bioanalyzer 2100 System (Agilent, Böblingen, Germany). A total of 1 μg RNA was transcribed to cDNA according to the protocol provided with the High Capacity cDNA Reverse Transcription kit (Life Technologies GmbH, Darmstadt, Germany). Gene expression profiling was performed using arrays of rat genome 230 2.0-type from Affymetrix (Affymetrix Inc., Santa Clara, CA, USA). Gene expression analyses were performed at the mRNA level by TaqMan low-density array (TLDA) (Invitrogen, Darmstadt, Germany). Pre-designed probe and primer sets for target genes were chosen from an online catalogue. Once selected, the sets were factory-loaded into the customized 384 wells of TLDA cards. Each TLDA card was configured into eight identical sets of 16 genes in triplicate. In all, 13 genes were chosen based on whole genome analysis. Each set of genes also contained two housekeeping/reference genes, *Ppia* and *Eif2b1*. Expression levels were measured in triplicate. Only the genes with reproducible amplification curves of the triplicates were analyzed and presented. TLDA cards were analyzed with RQ Manager Software (Invitrogen, Darmstadt, Germany) for automated data analysis. Gene expression values (RQ) were calculated based on the ∆∆Ct method [20]. A normal untreated animal tissue RNA pool was used as a calibrator and the *Ppia/Eif2b1* housekeeping gene were the references for normalization.

### Statistical analysis

The sample size calculation for testing the primary hypothesis (the gene expression of IL-6 in lung tissue is increased with BD HV_T_/LPEEP compared to BD LV_T_/OLPEEP) was based on effect estimates obtained from pilot studies as well as on previous measurements by our group (mean value and dispersion, respectively). Accordingly, we expected that a sample size of seven animals per group would provide the appropriate power (1-β = 0.8) to identify significant (α = 0.05) differences in IL-6 gene expression, considering an effect size d = 2.2, two-sided test and multiple comparisons (n = 3) (α* = 0.0167, α* Bonferroni adjusted). Data from non-survivors were excluded from further statistical analysis, except for two randomly chosen non-surviving animals from the BD HV_T_/LPEEP group included in the Affimetrix analysis. Thus, only surviving animals were evaluated.

The normality of the data and the homogeneity of variances were tested by means of the Shapiro-Wilk test and Levene’s median test, respectively. Both conditions were satisfied in all instances for physiological data; thus, one-way ANOVA was used followed by Holm-Sidak’s *post-hoc* test as required. Physiological data are expressed as mean ± standard deviation (SD).

Data from lung histology, expressed as median (25% to 75% interquartile range), were tested using Kruskal-Wallis followed by Tukey’s *post-hoc* test. Survival analysis was performed using the log-rank test.

Statistical analyses of physiological, histological and qPCR data, as well as survival analysis, were performed using SigmaPlot 11.0 (Systat Software GmbH, Erkrath, Germany). The level of significance was set at *P* <0.05.

Differential gene expression was analyzed based on log-linear mixed-model ANOVA, using the commercial software package SAS JMP7 Genomics, version 3.1 (SAS Institute, Cary, NC, USA). A type I error rate of alpha = 0.05 with Holm correction was taken as the level of significance. Pathways belonging to various cell functions were obtained from public external databases (Kyoto Encyclopedia of Genes and Genomes, KEGG). A Fisher’s exact test was performed to detect the significantly regulated pathways. PCR data were expressed as medians and 25% to 75% interquartile ranges and analyzed using one-way ANOVA on ranks and Tukey’s *post-hoc* test when appropriate. Statistical significance was set at *P* <0.05.

## Results

All animals in the NBD LV_T_/OLPEEP and BD LV_T_/OLPEEP groups survived, whereas in the NBD HV_T_/LPEEP group, one animal out of seven, and in the BD HV_T_/LPEEP group, three out of seven animals died. Mixing both NBD and BD groups together, LV_T_/OLPEEP resulted in improved survival compared to HV_T_/LPEEP (100% vs. 71.4%, *P* = 0.034).

At the end of six hours of ventilation, no significant physiological differences between NBD LV_T_/OLPEEP and NBD HV_T_/LPEEP were observed. Massive brain damage impaired oxygenation in BD HV_T_/LPEEP but not in BD LV_T_/OLPEEP (470.5 ± 26.7 vs. 321.6 ± 120.0, *P* = 0.005). Furthermore, after six hours, MAP was decreased in both BD groups compared to NBD groups (75.9 ± 10.6 mmHg (NBD LV_T_/OLPEEP End) *vs*. 64.6 ± 8.7 mmHg (BD LV_T_/OLPEEP End), *P* = 0.035, respectively, 72.4 + 20.3 mmHg (NBD HV_T_/LPEEP End) *vs.* 51.6 ± 14.1 (BD HV_T_/LPEEP End), *P* = 0.043) (Table 1).

**Table 1:** Physiological data at baseline and at the end of the experiment

	**BASELINE**		**END**		**END**	
	**LV**_**T**_**/OLPEEP n = 14**	**HV**_**T**_**/OLPEEP n = 14**	**NBD**		**BD**	
			**LV**_**T**_**/OLPEEP n = 7**	**HV**_**T**_**/LPEEP n = 6**	**LV**_**T**_**/OLPEEP n = 7**	**HV**_**T**_**/LPEEP n = 4**
V_T_ (ml/kg)	6.1 ± 0.3 ^+^	11.9 ± 0.2	5.9 ± 0.2 ^*^	12.2 ± 0.3	6.0 ± 0.3 ^#^	11.9 ± 0.2
PEEP (cmH_2_O)	5.0 ± 1.0 ^+^	2.0 ± 0.1	4.75 ± 1.0 ^*^	1.99 ± 0.0	5.2 ± 1.1 ^#^	2.0 ± 0.0
Pinsp (cmH_2_O)	12.6 ± 1.5 ^+^	16.5 ± 2.4	15.0 ± 2.2 ^*^	18.8 ± 4.8	15.0 ± 0.9 ^#^	23 ± 7.6
Pmean (cmH_2_O)	7.9 ± 1.1 ^+^	6.0 ± 1.1	8.3 ± 1.1	8.7 ± 2.0	9.0 ± 0.9	10.3 ± 2.7
RR (bpm)	100.3 ± 2.9 ^+^	31.4 ± 2.4	108.1 ± 5.3 ^*‡^	30.6 ± 4.2	102.3 ± 4.4 ^#^	29.2 ± 3.8
Estat, RS (cmH_2_O.ml^−1^)	2.41 ± 0.4	2.39 ± 0.4	3.42 ± 0.9	2.74 ± 0.9	3.06 ± 0.4	3.44 ± 1.2
Estat, L (cmH_2_O.ml^−1^)	1.83 ± 0.4	1.9 ± 0.4	2.59 ± 1.0	2.23 ± 0.9	2.29 ± 0.4	2.90 ± 1.2
Estat, CW (cmH_2_O.ml^−1^)	0.58 ± 0.2	0.37 ± 0.2	0.49 ± 0.3	0.31 ± 0.1	0.76 ± 0.3	0.50 ± 0.2
PaO_2_/FiO_2_	439.2 ± 47.4	463.8 ± 32.5	458.8 ± 44.9	410.2 ± 129.9	470.5 ± 26.7 ^#^	321.6 ± 120.0
PaCO_2_ (mmHg)	47.5 ± 9.7	41.2 ± 8.6	47.6 ± 12.1	37.5 ± 5.6	39.0 ± 4.5	37.35 ± 5.6
pHa	7.34 ± 0.0	7.34 ± 0.0	7.34 ± 0.1 ^‡^	7.38 ± 0.0	7.42 ± 0.0	7.41 ± 0.1
HR (bpm)	255.4 ± 40.6	284.0 ± 42.7	230.5 ± 60.0 ^‡^	262.5 ± 31.1	292.5 ± 52.6	314.0 ± 90.9
MAP (mmHg)	63.2 ± 11.4	70.5 ± 8.8	75.9 ± 10.6 ^‡^	72.4 + 20.3 ^$^	64.6 ± 8.7	51.6 ± 14.1

BD, brain damage; Estat,CW, chest wall static elastance; Estat,L, lung static elastance; Estat,RS, respiratory system static elastance; HR, heart rate; HV_T_/LPEEP, six hours of high tidal volume ventilation with low PEEP, no brain damage; LV_T_/OLPEEP, six hours of low tidal volume ventilation with open lung positive end-expiratory pressure; MAP, mean arterial pressure; NBD, no brain damage; PaCO_2_, partial pressure of carbon dioxide; PaO_2_/FiO_2_, ratio of partial pressure arterial oxygen and fraction of inspired oxygen; PEEP, positive end-expiratory pressure; pHa, arterial pH; Pinsp, end-inspiratory pressure; RR, respiratory rate; V_T_, tidal volume.

Values are mean ± standard deviation.

+: *P* <0.05 LV_T_/OLPEEP BL vs. HV_T_/LPEEP BL.

*: *P* <0.05 NBD LV_T_/OLPEEP End *vs*. NBD HV_T_/LPEEP End.

#: *P* <0.05 BD LV_T_/OLPEEP End *vs*. BD HV_T_/LPEEP End.

‡: *P* <0.05 NBD LV_T_/OLPEEP End *vs*. BD LV_T_/OLPEEP End.

$: *P* <0.05 NBD HV_T_/LPEEP End *vs.* BD HV_T_/LPEEP End.

The BD LV_T_/OLPEEP group received more fluid than the NBD HV_T_/LPEEP group over the experimental period of six hours (NBD LV_T_/OLPEEP: 45 ± 6.8 ml; NBD HV_T_/LPEEP: 38.25 ± 3.8 ml; BD LV_T_/OLPEEP: 54.5 ± 8.1 ml (*P* <0.001 vs. NBD HV_T_/LPEEP); BD HV_T_/LPEEP 45.5 ± 9.4 ml).

Total lung injury score was higher in BD compared to NBD in HV_T_/LPEEP (4.5 (3.75/6.25) vs. 8 (6.5/8.75), *P* = 0.026) but not in LV_T_/OLPEEP.

LV_T_/OLPEEP reduced inflammation in both NBD and BD (0 (0/0) (NBD LV_T_/OLPEEP) vs. 2 (0.75/2.5) (NBD HV_T_/LPEEP), *P* = 0.01, respectively; 0 (0/0.25) (BD LV_T_/OLPEEP) *vs*. 2.5 (2/3) (BD HV_T_/LPEEP), *P* <0.001), while over-inflation was observed only in BD HV_T_/LPEEP (1 (1/1.25) (BD LV_T_/OLPEEP) *vs*. 2.5(2/3) (BD HV_T_/LPEEP), *P* = 0.029) (Table 2).

**Table 2:** Histological lung injury score

	**NBD**		**BD**	
	**LV**_**T**_**/OLPEEP**	**HV**_**T**_**/LPEEP**	**LV**_**T**_**/OLPEEP**	**HV**_**T**_**/LPEEP**
Total	2.5 (1.75/3)†	4.5 (3.75/6.25)‡	3 (3/3.25)*	8 (6.5/8.75)
Haemorrhage	0 (0/0)	0 (0/0)	0 (0/0)	0 (0/0.75)
Inflammation	0 (0/0)†	2 (0.75/2.5)	0 (0/0.25)*	2.5 (2/3)
Oedema	0 (0/0)†#	1 (0/1)	1 (0/1)	1.5 (1/2)
Atelectasis	1 (0.75/1)	0 (0/1)	1 (1/1)	0 (0/0.75)
Over-inflation	1 (1/1.25)	2 (1/2.25)	1 (1/1.25)*	2.5 (2/3)

All values presented as median and 25% to 75% interquartile ranges.

BD; brain damage; NBD, no brain damage; HV_T_/LPEEP, six hours of high tidal volume ventilation with low PEEP; LV_T_/OLPEEP, six hours of low tidal volume ventilation with open lung positive end-expiratory pressure;

†: *P* <0.05 NBD LV_T_/OLPEEP vs. NBD HV_T_/LPEEP.

*: *P* <0.05 BD LV_T_/OLPEEP *vs*. BD HV_T_/LPEEP.

#: *P* <0.05 NBD LV_T_/OLPEEP *vs*. BD LV_T_/OLPEEP.

‡: *P* <0.05 NBD HV_T_/LPEEP *vs*. BD HV_T_/LPEEP.

**Figure 1:**
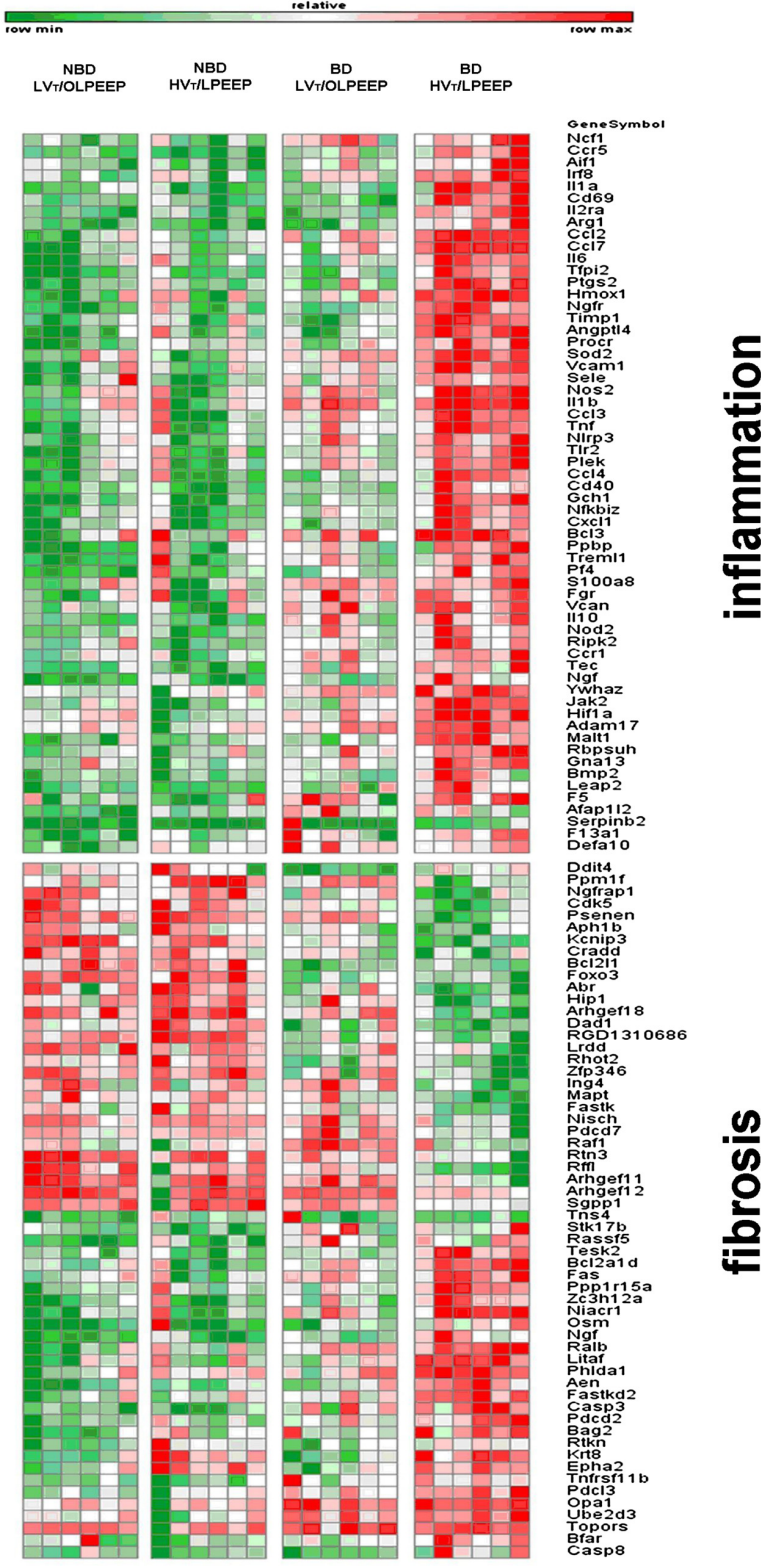
**Heat map for gene expression.** Genes were clustered using MAPPFinder. Gene ontologies of inflammation and fibrosis are shown. Red color indicates up-regulation, green color indicates down-regulation relative to the mean overall four treatment groups, while color intensity corresponds to the fold-change amplitude. BD, brain damage; HV_T_/LPEEP, six hours of high tidal volume ventilation with low PEEP, no brain damage; LV_T_/OLPEEP, six hours of low tidal volume ventilation with open lung positive end-expiratory pressure; NBD, no brain damage.

In both NBD and BD, LV_T_/OLPEEP reduced the number of expressed genes compared to HV_T_/LPEEP (183 *vs*. 1,073 genes and 393 *vs.* 1,974 genes, respectively). Overall, in both NBD and BD, 28 genes simultaneously showed different expression between LV_T_/OLPEEP and HV_T_/LPEEP (Figure 1). The complete dataset, including normalized and raw data, is available at the GEO repository with accession number (GSE52142).

**Figure 2:**
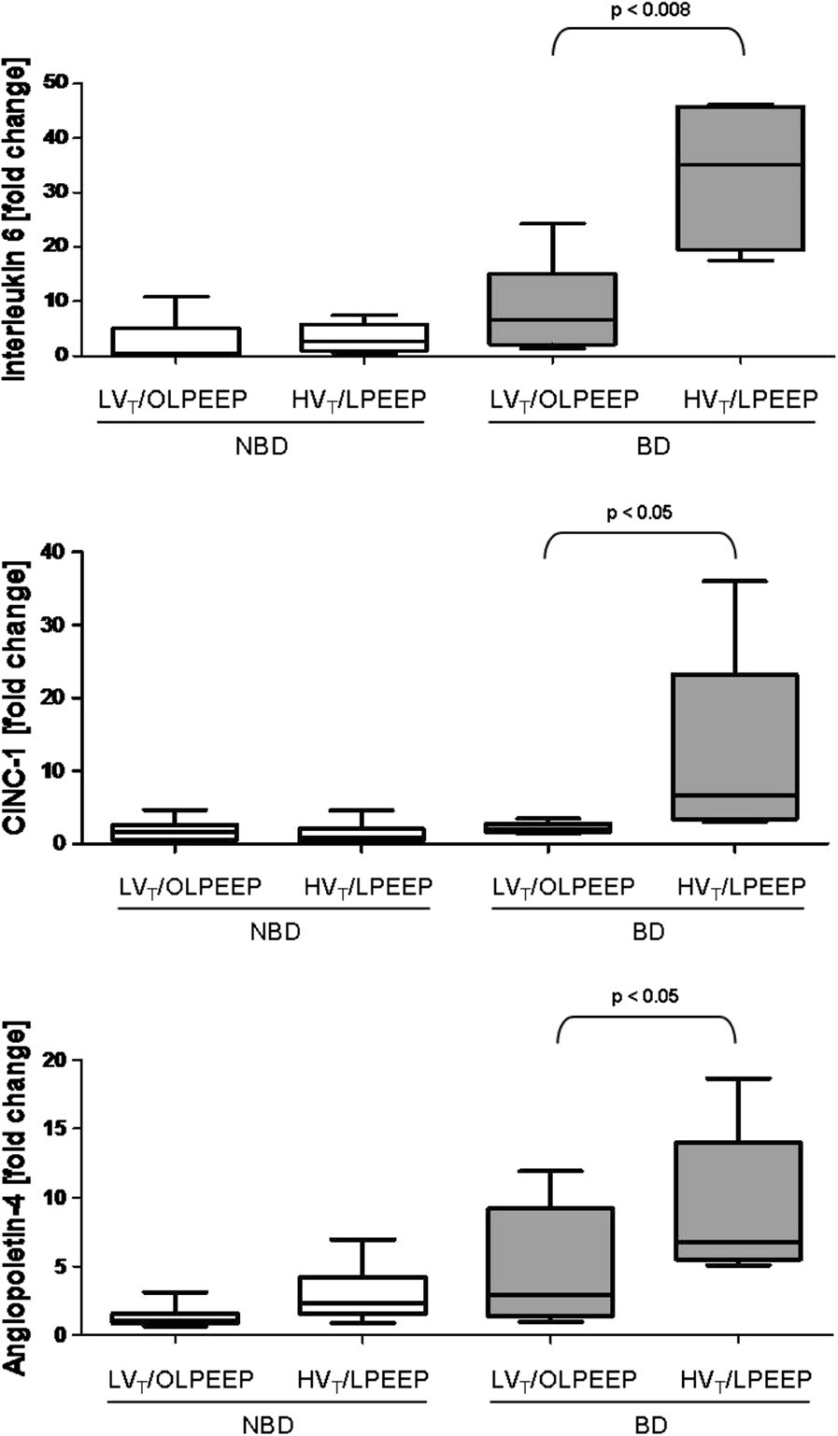
**Polymerase chain reaction of lung tissue.** All values are presented as median and 25% to 75% interquartile ranges. *P*-values above brackets showed significant difference (*P* <0.05). BD, brain damage; HV_T_/LPEEP, six hours of high tidal volume ventilation with low PEEP, no brain damage; LV_T_/OLPEEP, six hours of low tidal volume ventilation with open lung positive end-expiratory pressure; NBD, no brain damage.

In brain damaged animals, HV_T_/LPEEP led to a significant increase of interleukin 6 (IL-6) (6.7 (3.5/10.5) (BD LV_T_/OLPEEP) *vs*. 35.2 (23.6/45.2) (BD HV_T_/LPEEP), *P* = 0.008), cytokine-induced neutrophil chemoattractant 1- (CINC-1) (2.0 (1.7/2.5) (BD LV_T_/OLPEEP) *vs*. 6.7 (4.2/16.0) (BD HV_T_/LPEEP), *P* = 0.004) and angiopoietin 4 (2.9 (1.9/5.6) (BD LV_T_/OLPEEP) *vs*. 6.8 (5.9/11.1) (BD HV_T_/LPEEP), *P* = 0.043) mRNA expressions in qPCR compared to BD LV_T_/OLPEEP, while no significant differences were detected in the absence of brain damage (Figure 2). No significant differences between BD LV_T_/OLPEEP and BD HV_T_/LPEEP were found in tumor necrosis factor (TNF)-α, monocyte chemotactic protein (MCP) 1 and 5, E-selectin, vascular cell adhesion protein 1, heme oxygenase-1, nitric oxide synthase-2, hypoxia inducible factor-1α and caspase-1.

## Discussion

In the present study, we investigated the effects of a lung-protective ventilatory strategy by using low V_T_ and PEEP titrated to the minimal static elastance of the respiratory system (LV_T_/OLPEEP) compared to high V_T_ and low PEEP (HV_T_/LPEEP) on lung morphofunction and whole genome differences in the presence and absence of massive brain damage. Following a recruitment maneuver, PEEP set at minimal elastance is thought to represent open lung PEEP according to the mathematical model proposed by Hickling [21]. In NBD, both LV_T_/OLPEEP and HV_T_/LPEEP did not affect arterial blood gases, as well as whole genome expression changes and real-time PCR. In BD, LV_T_/OLPEEP compared to HV_T_/LPEEP reduced lung damage according to histology, genome analysis and real-time PCR with significantly lower interleukin (IL-6), CINC-1 and angiopoietin-4 expressions.

Protective mechanical ventilation with low V_T_ has been associated with reduced mortality and/or morbidity in patients with or without ARDS [10,11,22]. Furthermore, low V_T_ with high PEEP compared to high V_T_ and low PEEP has been found to prevent postoperative pulmonary complications in patients with non-injured lungs [23]. However, protective ventilation strategies using low V_T_ and high PEEP are not usually applied in patients with massive brain injury, due to the risk of hypercapnia and reduced cerebral perfusion [24]. In this line, patients with brain damage are usually ventilated with V_T_ higher than 6 mL/kg and low PEEP [2], even though it may be associated with lung damage, inflammation and, ultimately, organ failure [25].

In this study, NBD HV_T_/LPEEP was associated with higher total histological damage compared to NBD LV_T_/OLPEEP, which was mainly related to inflammation and edema. High volume at end-inspiration is well known to promote alveolar distension, disruption of pulmonary epithelium and endothelium, recruitment of pro-inflammatory cells in the lung tissue and the induction of edema eventually resulting in hypoxia [12]. Protti *et al*. [26] demonstrated a lung strain (the ratio between tidal volume and the functional residual capacity) threshold of higher than 1.5 to 2.0 to be associated with pulmonary dysfunction, emphysematous change, alveolar hemorrhage, hyaline membrane formation and lung weight gain in previously healthy lungs in pigs.

The induction of brain damage led to a higher total histology damage score of BD HV_T_/LPEEP compared to both NBD HV_T_/LPEEP and BD LV_T_/OLPEEP caused primarily by over-inflation and the amount of pro-inflammatory cells in lung parenchyma (Table 2).

This aggravation of lung histological impairment is in line with findings from other groups [27,28], implying that brain damage sensitizes the lung for the potentially deleterious effects of high intrathoracic gas volumes [11] and intratidal re- and de-recruitment [29].

No statistically significant differences in lung mechanics, gas exchange and hemodynamics after the six-hour experimental period between NBD LV_T_/OLPEEP and NBD HV_T_/LPEEP were found showing the comparatively minor functional effects of stress and strain below threshold *in vivo* as also shown by Protti *et al*. [26]. Conversely, in BD, we found a significant impairment of oxygenation in HVT/LPEEP compared to LVT/OLPEEP. Furthermore, MAP was reduced in both BD groups.

As we did not measure cardiac output or perform morphological studies using echocardiography, we can only speculate whether these hemodynamic changes indicate right ventricular failure caused by insufficient end-expiratory lung volume [30] and inflammation-mediated increased afterload [31].

Gene ontology analysis allocated genes showing significant changes in inflammatory and, in a much lesser degree, to profibrotic pathways. In general, we found more changed genes than previously described [32-35].

Comparing LV_T_/OLPEEP with HV_T_/LPEEP, RT-PCR of lung parenchyma showed increased IL-6, CINC-1 and angiopoietin-4 expressions in BD but not in NBD (Figure 2). In this context, previous studies indicate that IL-6 and CINC-1, pro-inflammatory cytokines, contribute to ventilator induced-lung injury [36-38]. In the present study, the increase in IL-6 and CINC-1 expressions with HV_T_/LPEEP may be related to mechanotransduction in lung tissue by increased distortion of the alveolar-capillary barrier [37]. Moreover, HV_T_/LPEEP the increased neutrophil infiltration and lung edema may be associated with increased angiopoietin-4 [39].

Several mechanisms may explain the inflammatory potentiation of brain injury, going beyond the impact of high end-tidal intrathoracic gas volumes [2,9]. McKeating *et al*. showed that the injured brain itself is a potent source of pro-inflammatory cytokines which are released in the circulation [40]. Additionally, we noted a distinctive and rapid two-fold increase in MAP from baseline during the induction of brain injury in the BD LV_T_/OLPEEP and BD HV_T_/LPEEP groups (data not shown). Brain injury causes a potent sympathoadrenergic response known as the Cushing response, which has pro-inflammatory properties [27,41] and causes hypertensive crisis, pulmonary venoconstriction and, consecutively, elevation of pulmonary artery peak pressure [31,42], resulting in a marked distortion of the alveolar-capillary membrane [27,43]. In addition, the protective anti-inflammatory effect of vagus nerve efferents is lost in massive brain injury [5], thus aggravating the systemic inflammatory response.

The minimized lung morpho-functional changes and inflammatory responses observed in BD brain damaged animals ventilated with low tidal volume and PEEP set to minimal static elastance compared to animals ventilated with high tidal volume and low PEEP may provide some pathophysiological insights explaining the clinical results from Mascia *et al*. [44]

In this randomized controlled clinical trial performed in brain dead organ donors examining the effects of a lung protective ventilatory strategy consisting of tidal volumes of 6 to 8 mL/kg of predicted body weight, high PEEP and precautions to preclude de-recruitment resulted in an increased number of eligible and harvested lungs compared with a high tidal volume/low PEEP strategy.

### Limitations

The current study has several limitations that need to be addressed. First, we aimed to simulate clinically relevant ventilator settings. We did not include control groups with neither ventilator strategies resulting in high end-tidal intrathoracic volumes (like HV_T_/OLPEEP), since they are not recommended in clinical practice. Therefore, we were unable to identify whether the reduction of the V_T_ or the optimization of the functional residual capacity with an optimized PEEP caused the beneficial effects of LVT/OLPEEP, since a group with high V_T_ and OL/PEEP was not analyzed. Second, we chose to maintain a constant PaCO_2_ by decreasing the respiratory rate in the high V_T_ group rather than adding instrumental dead space. Third, we did not use catecholamines but a rather liberal fluid management to prevent hypotension and avoid possible interaction in modulating inflammatory response, organ function and gene expression [18,45-47]. Fourth, we did not assess whether the LV_T_/OLPEEP strategy can protect other organs except the lung from the pro-inflammatory effects of massive brain damage. Fifth, we studied a specific model of severe brain damage [5] and do not know if our results would apply to other models [10,23] or species [48]. We did not study neurological parameters, but focused on the lung rather than on the potential impact of the open lung strategy on brain damage. Therefore, further studies will be required to better define the suitable ventilatory strategy in the setting of massive brain damage. Sixth, the severity of brain damage in our model was not quantified and its potential evolution with different mechanical strategies was not analyzed. Seventh, even though the gene expression profile through genome analysis demonstrated significant differences in the number of regulated genes between both ventilation strategies, the biological significance of these changes in terms of lung tissue damage requires clarification. Nonetheless, confirmatory qPCR analyses for selected genes that previously had been suggested to be implicated in ventilator induced lung injury [36–38] were significantly higher in the HVT/LPEEP group. Whether the increased mRNA expression in turn translates into higher protein expression was not studied and does demand further investigation. Similarly, the contribution of IL-6, CINC-1 and angiopoeitin-4 to the different extent of lung tissue damage in both groups needs to be better evaluated. Nevertheless, these analyses may be considered a starting point in the evaluation of the impact of different ventilator strategies in the presence of severe brain damage.

## Conclusion

In the present study, low tidal volume ventilation with open lung PEEP minimized lung morpho-functional changes and inflammation in the presence of massive brain injury compared to high tidal volume ventilation with low PEEP.

## Key messages

• The combination of high tidal volume ventilation and massive brain damage causes a proinflammatory reaction in pulmonary parenchyma with deterioration in histological analysis and impairment of gas exchange whereas high tidal volume ventilation alone did not.

• Low tidal volume ventilation with PEEP titrated to the minimal static elastance of the respiratory system could alleviate the inflammatory stimulus of massive brain damage.

## Abbreviations

ARDS: Acute respiratory distress syndrome; BD: Brain damage; cDNA: Complementary deoxyribonucleic acid; CINC-1: Cytokine-induced neutrophil chemoattractant 1; CPP: Cerebral perfusion pressure; DNA: Deoxyribonucleic acid; Estat,CW: Static elastance of the chest wall; Estat,L: Static lung elastance; Estat,RS: Static elastance of the respiratory system; FiO2: Fraction of inspired oxygen; HR: Heart rate; HVT/LPEEP: High tidal volume ventilation with low positive end-expiratory pressure; i.p.: Intraperitoneal injection; I:E: Inspiratory:expiratory ratio; ICU: Intensive care unit; IL-6: Interleukin 6; kg: Kilogram; LVT/OLPEEP: Low tidal volume ventilation with positive end-expiratory pressure titrated to minimal static elastance; MAP: Mean arterial pressure; mL: Millilitre; mRNA: Messenger ribonucleic acid; NBD: No brain damage; PaCO2: Partial pressure of carbon dioxide; PEEP: Positive end-expiratory pressure; Pes: Esophageal pressure; Pinsp: End-inspiratory plateau pressure; Ptrach: Tracheal pressure; qPCR: Quantitative polymerase chain reaction; RR: Respiratory rate; SD: Standard deviation; TLDA: TaqMan low density array; VALI: Ventilator-associated lung injury; VT: Tidal volume; ΔPes: Difference between end-inspiratory and end-expiratory esophageal pressure; ΔPtrach: Difference between end-inspiratory and end-expiratory tracheal pressure.

## Competing interests

The authors declare that they have no competing interests.

## Authors’ contributions

JK, CT, PP, PRMR and TL participated in the study design. JK, CT, MH, BY and TL performed the study. JK, CT, MH, CS, BY and TL processed the data and performed the statistical analysis. JK, CT, PP, PRMR, BY and TL wrote the manuscript. CS and MH critically revised the manuscript for important intellectual content. All authors read and approved the final manuscript.
